# Comparison of body fat percentage assessments by bioelectrical impedance analysis, anthropometrical prediction equations, and dual-energy X-ray absorptiometry in older women

**DOI:** 10.3389/fnut.2022.978971

**Published:** 2022-12-21

**Authors:** María Consuelo Velázquez-Alva, María Esther Irigoyen-Camacho, Marco Antonio Zepeda-Zepeda, Itzam Rangel-Castillo, Isabel Arrieta-Cruz, Luciano Mendoza-Garcés, Antonio Castaño-Seiquer, Javier Flores-Fraile, Roger Gutiérrez-Juárez

**Affiliations:** ^1^Department of Health Care, Metropolitan Autonomous University, Unit Xochimilco, Mexico City, Mexico; ^2^National Institute of Geriatrics, Ministry of Health, Mexico City, Mexico; ^3^Faculty of Dentistry, University of Seville, Seville, Spain; ^4^Department of Surgery, Faculty of Medicine, University of Salamanca, Salamanca, Spain; ^5^Department of Biomedical Sciences, Faculty of Higher Studies Zaragoza, School of Medicine, National Autonomous University of Mexico, Mexico City, Mexico

**Keywords:** aging, body fat, anthropometric, bioelectrical impedance, DXA (dual X-ray absorptiometry), validation studies

## Abstract

**Background:**

Individuals with high body fat have a higher risk of mortality. Numerous anthropometric-based predictive equations are available for body composition assessments; furthermore, bioelectrical impedance analysis (BIA) estimates are available. However, in older adults, the validity of body fat estimates requires further investigation.

**Objective:**

To assess the agreement between percentage body fat (BF%) estimates by BIA and five predictive equations based on anthropometric characteristics using dual X-ray absorptiometry (DXA) as reference method. A secondary objective was to identify whether excluding short-stature women improves the agreement of BF% estimates in a group of community-dwelling, older Mexican women.

**Methods:**

A concordance analysis of BF% was performed. A total of 121 older women participated in the study. Anthropometric information, BIA, and DXA body composition estimates were obtained. Five equations using anthropometric data were evaluated in order to determine body fat percentage (BF%) using DXA as reference method. Paired *t*-test comparisons and standard error of estimates (SEE) were obtained. The Bland-Altman plot with 95% limits of agreement and the concordance correlation coefficient (CCC) were used to evaluate the BF% prediction equations and BIA estimates.

**Results:**

The mean age of the study participants was 73.7 (±5.8) years old. BIA and the anthropometric based equations examined showed mean significant differences when tested in the entire sample. For the taller women (height > 145 cm), no significant difference in the paired comparison was found between DXA and BIA of BF% estimates. The mean BF% was 40.3 (±4.8) and 40.7 (±6.2) for DXA and BIA, respectively. The concordance between methods was good (CCC 0.814), (SEE 2.62). Also, in the taller women subset, the Woolcott equation using waist-to-height ratio presented no significant difference in the paired comparison; however, the error of the estimates was high (SEE 3.37) and the concordance was moderate (CCC 0.693).

**Conclusion:**

This study found that BIA yielded good results in the estimation of BF% among women with heights over 145 cm. Also, in this group, the Woolcott predictive equation based on waist circumference and height ratio showed no significant differences compared to DXA in the paired comparison; however, the large error of estimates observed may limit its application. In older women, short stature may impact the validity of the body fat percentage estimates of anthropometric-based predictive equations.

## Introduction

In 2019, the global population of older adults aged 60 and over was nearly 1 billion people, representing 13.2% of the total population. By 2050, their number is expected to reach 2.1 billion, 2.5 times more than in 1980 (382 million). By 2050, United Nations projections estimate that there will be twice as many older adults as children under the age of five. Most older adults live in middle-income countries (1). With an aging population, it is particularly difficult to adequately respond to related epidemiological changes, such as the increasing rate of chronic non-communicable diseases (NCDs). According to Global Burden of Disease (GBD) estimates in 2015, 28.9% of GBD was attributable to people over 60 years of age, and NCDs accounted for 86.8% of the total burden of disease (2, 3). In European countries the prevalence of obesity has increased rapidly in the last 40 years, particularly among adults aged 60–74 years (4). The results of a study in China showed that more than half of the Chinese aged 70 years or older have obesity-associated multimorbidity, which has become a major public health problem in this country (5, 6). Older adults will develop obesity and multiple chronic diseases (Type-2 diabetes mellitus, cardiovascular and cerebrovascular diseases, high blood pressure, dyslipidemias, metabolic syndrome, and abdominal adiposity) generating a reduction in the quality of life. In addition, obesity is also associated with greater disability and worsening of non-communicable chronic diseases (NCDs) (7, 8). In Mexico there is a high prevalence of obesity, mainly, in sectors with greater poverty and vulnerability. According to the Mexican National Survey of Health and Nutrition (ENSANUT), in the range of women aged 60–69 years, there was an increase in the prevalence of obesity from 41.0% in 2012 to 45.9% in 2018 (9).

The method most widely used to estimate obesity is the body mass index (BMI) (kg/m^2^), because it is simple and inexpensive and is the basis for the World Health Organization (WHO) criteria of overweight (25 ≤ BMI < 30) and obesity (BMI ≥ 30) (10). However, for a given BMI, body fat percentage changes with age, and the form of this change is different according to sex, ethnicity, and individual differences (11). Changes in body composition due to aging have led to the proposal of different cut-off points for defining underweight and overweight in older adults. Lipschitz considers that older adults with BMI ≤ 22 kg/m^2^ are underweight and those with BMI > 27 kg/m^2^ are overweight or obese (12).

Additionally, height plays a very important role in determining BMI. Changes in height that occur during aging will impact the BF estimates using BMI (13). The decrease in height occurs mainly due to the following factors: reduction of the plantar arch, increase in the curvature of the spine, vertebral compression, shape of the vertebral discs, loss of muscle tone and inadequate posture habits as well as due to injuries and diseases that affect the joints and the musculoskeletal system (14). After 50 years of age, men’s height decreases between 0.08% and 0.10%, while women’s height declines between 0.12 and 0.14% per year, sharpening after 70 years of age (14, 15). In China, the height of women decreases by 3.8 cm every 10 years from the age of 40, while in Indonesia it decreases by 0.6 cm per year for women 60 years and older (16). Age-related changes in height have been associated with health problems (17, 18). Mexican women of short stature and over 50 years of age had an increased risk of obesity (OR = 1.84) compared to women without this condition (19).

To obtain a complete nutritional evaluation of older adults, body composition should be considered for both the nutritional diagnosis (risk of malnutrition or malnutrition) (20, 21) as well as to determine the different body compartments and assess more precisely if the patient presents obesity (22), sarcopenia (decrease in appendicular skeletal muscle mass) (23) or osteoporosis (decrease in bone mineral density) (24).

Dual X-ray absorptiometry (DXA) is frequently used as the gold standard for evaluating body composition prediction methods. DXA estimates are at a molecular level and identify three body components: bone mineral content (BMC), lean mass (LM), and fat mass (FM). This technique has shown good agreement compared with more sophisticated techniques (25).

Bioelectrical impedance analysis (BIA) is increasingly used to evaluate body composition. BIA safe, BMI is simple to apply, non-invasive, and inexpensive as it avoids radiation exposure (26). Based on the electrical properties of the body, BIA determines the resistance resulting from an electrical current passing through the body. It considers the subject’s weight, height, and age to estimate, the total body’s water, and applies specific equations (Siri or Brosek) (27, 28) in order to determine the BF%. BIA is a doubly indirect method of assessing body composition. Since it is based on factors such as type of device, water distribution, hydration status, weight, and height, BIA estimates may vary (29).

Studies of comparison of DXA and BIA for body composition assessment are scarce in older adults. There is considerable interest in the field of body composition for developing and properly validating equations based on anthropometric measurements so as to determine lean body mass, fat mass percentage, and fat content in wide population groups without having to use technologies such as DXA (30). Currently, in older adults, there is no agreement on whether equations based on anthropometric data can successfully be used in clinical practice or public health settings. There are conflicting results on the validity of predicting equations available based on anthropometric characteristics (31). Some studies have shown good agreement while others found low concordance and biased estimates when comparing predictive equations with DXA (32, 33).

The objective of the study was to assess the agreement between percentage body fat (BF%) estimates by BIA and five predictive equations based on anthropometric characteristics using DXA as reference method. A secondary objective was to identify whether excluding short-stature women improves the agreement of BF% estimates in a group of community-dwelling, older Mexican women.

## Materials and methods

### Study design

The current study has a cross-sectional design. The study group was selected from attendees of a sports and social entertainment facility in Southeast Mexico City, between April and July of 2019. This facility has governmental support and is free of charge for people over 60 years old. There are several activities that the attendees can engage in, such as dancing classes, needle knitting, and singing lessons (chorus). Also, a gym is available, and attendees may participate in gymnasia, physical conditioning, spinning, yoga, Tai Chi, and similar classes.

To enroll participants in the study, we placed an ad at the entrance and registered those who were interested in receiving nutritional assessment and have a DXA evaluation. All the procedures were free of charge for the facility members. The eligibility criteria of the study were the following: women over 60 years old, capable of independent mobility (not using a wheelchair), who were under medical treatment and supervision if they had NCDs. The women who were willing to participate in the study signed an informed consent letter. Among the exclusion criteria were women who have a recent history of falls and fractures or recent hospital admissions (within the last 6 months), those with serious medical conditions (cardiovascular or cerebrovascular disease, respiratory failure, liver failure, Parkinson’s disease, advanced diabetic neuropathy, rheumatoid arthritis, and cognitive impairment) were also excluded from the study as well as those with signs of edema, physical disability, and those wearing an orthopedic prosthesis that could alter their body composition results. The study’s goals and evaluation procedures were individually described in a detailed form to each participant. All subjects signed an informed consent letter in which the goals and procedures of the study were fully described. This study was conducted in accordance with the ethical standards of the Helsinki Declaration of Ethical Principles for Medical Research Involving Human Subjects. Recruitment and data collection took place between April and July 2019. The protocol was registered and approved by the Division of Biological and Health Sciences and the Ethics Committee of the Universidad Autónoma Metropolitana Unidad Xochimilco (DCBS.CD, approval CD.52.17).

### Anthropometry

Body weight and height measurements were taken by a certified dietitian (International Certification in Kinanthropometry, Isak Level 1) using the recommended techniques and procedures (34). A senior researcher supervised the anthropometric evaluation. Body weight and height were measured using a portable, electronic digital scale, equipped with a built-in stadiometer with a resolution of 0.1 kg and 0.1 cm, respectively, according with Lohman et al.’s specifications (35). The waist circumference was measured with a fiberglass tape and was reported in centimeters. The anatomic landmarks used to measure waist circumference were the midpoint between the lower rib and the iliac crest T, crest. This is considered the ideal place to perform the procedure. BMI was calculated dividing body weight (kg) by the square of height (m) and expressed in kg/m^2^. Using the WHO criteria, the participants were classified according to BMI in four groups: low (BMI < 18.5 kg/m^2^); normal (BMI 20 –24.9 kg/m^2^); overweight (BMI 25.0–29.9 kg/m^2^) and obese (BMI > 30 kg/m^2^) (36). Additionally, a BMI classification especially design for older adults was applied in the study group. The Lipschitz cut-off points proposed for individuals older than 65 years were also applied: underweight (BMI < 22 kg/m^2^), eutrophic BMI (22–27 kg/m^2^) and excess weight (BMI > 27 kg/m^2^) (12).

### Body composition assessment

#### Dual-energy X-ray absorptiometry (DXA)

Participants were required to wear light sport clothing free of metal zippers and metal decorations, jewelry (watches, earrings, necklaces, and rings), hairpins and coins, keys, to avoid interference with DXA measurements. Whole body DXA scans were carried out following the manufacturer’s instructions by a laboratory technician with experience, using the Hologic Discovery QDR Series DXA equipment. The technician inspected each scan image and performed the necessary corrections to ensure reliable and high-quality results. The DXA equipment was calibrated daily in the morning with a phantom prior to the actual measurements. Values of total BF expressed in grams and percentage, as well as fat free mass (in grams) were determined directly with DXA. To perform the scan, each participant was asked to lay down on the equipment table in a supine position along their longitudinal axis, using the middle line as a reference. Each participant was asked to keep the toe tips in close contact while the scans were performed. The women’s hands were kept in a prone position within the scan field of the equipment. While the body scan was being performed, participants were asked to stay still. Whole body scans had a mean length of 6 min per person.

#### Bioelectric impedance analysis (BIA)

A multiple frequency equipment with a current between 100 and 500 μA was used. The device was equipped with eight tactile electrodes (four in the platform, to make feet connect, and four on each of the two handles, to connect the hand fingers in order to ensure passage of the electric current. The women fasted 8–12 h prior to each BIA or DXA measurement. The evaluations were performed in the morning. Each person was told to avoid over-hydration and to avoid performing strenuous exercise. Each participant emptied their bladder prior to the BIA or DXA test. Participants were asked to take off their shoes and to maintain an orthostatic position (standing up) during BIA measurements.

#### Equations to predict body fat percentage

The number of equations available in the literature to estimate body fat is large; therefore, it is not practical to test all of them. We selected five equations for the prediction of BF%. The criteria for selecting these equations were: (1) Equations developed including adults and older adults in the study group; (2) Equations including white or Hispanic ethnic groups in the development process; (3) An adequate sample size; (4) Only requiring anthropometric, sex, and age data to obtain the BF% estimates; (4) Whether these equations were used in other tests with good results (cross-validation study). Table 1 presents the characteristics the five equations selected.

**TABLE 1:** Selected equations presenting age, body mass index, body fat percentage, coefficient of determination and measurement error in women.

References	Equations	*n*	Age (years) mean/range	BMI (kg/m^2^) mean/range	Body fat %	*R* ^2^	SEE/ RMSE
Deurenberg et al. (54)	%*BF* = 1.20×*BMI* + 0.23×*age*−10.8× *sex*−5.4	708	7–83	13.9–40.9 kg/m^2^	Group A: 24.7 Group B^a^: 25.7	0.79	4.1
Gallagher et al. (11)	$\%BF=64.5-848\text{×}\;(\frac{1}{BMI})+0.79\text{×}age$	1,013	African American: 56.2 ± 16.8 White: 48.8 ± 17.6 Asian: 39.3 ± 15.9	African American: 27.1 ± 4.3 White: 24.5 ± 4.5 Asian: 23.2 ± 3.9	African American: BMI < 18.5: 23% BMI ≥ 25: 35% BMI ≥ 30: 41% White: BMI < 18.5:25% BMI ≥ 25: 38% BMI ≥ 30: 43% Asian: BMI < 18.5:26% BMI ≥ 25: 36% BMI ≥ 30: 41%	0.86	4.98
Woolcott and Bergman (55)	$\%BF=64-20\text{×}\;(\frac{height}{waist})+12\text{×}sex$	NHANES 1999–2004 6,261 NHANES 2005–2006 1,700	NHANES 1999–2004 47.2 ± 0.3 NHANES 2005–2006 43.3 ± 0.3	NHANES 1999–2004 28.2 ± 0.1 NHANES 2005–2006 28.7 ± 0.3	NHANES 1999–2004 30.8 ± 0.3 NHANES 2005–2006 31.2 ± 0.6	0.70–0.49	3.91–4.01
Woolcott and Bergman waist/height (55)	$\%BF=-5+58\text{×}\left(\frac{waist}{height}\right)+11\times sex$	NHANES 1999–2004 6,261 NHANES 2005–2006 1,700	NHANES 1999–2004 47.2 ± 0.3 NHANES 2005–2006 43.3 ± 0.3	NHANES 1999–2004 28.2 ± 0.1 NHANES 2005–2006 28.7 ± 0.3	NHANES 1999–2004 30.8 ± 0.3 NHANES 2005–2006 31.2 ± 0.6	0.67–0.48	4.12–4.07
Woolcott and Bergman height^3^ / waist × weight (55)	$\%BF=44-230\text{×}\;(\frac{height^{3}}{waist\times weight})+12\times sex$	NHANES 1999–2004 6,261 NHANES 2005–2006 1,700	NHANES 1999-2004 47.2 ± 0.3 NHANES 2005–2006 43.3 ± 0.3	NHANES 1999–2004 28.2 ± 0.1 NHANES 2005–2006 28.7 ± 0.3	NHANES 1999–2004 30.8 ± 0.3 NHANES 2005-2006 31.2 ± 0.6	0.75–0.61	3.60–3.51

^a^Group B: cross-validation group. BMI, body mass index; %BF, percentage of body fat; *R*^2^, coefficient of determination; SEE, standard error of the estimate; RMSE, root mean squared error; NHANES, National Health and Nutrition Examination Survey.

#### Sample size

**FIGURE 1:**
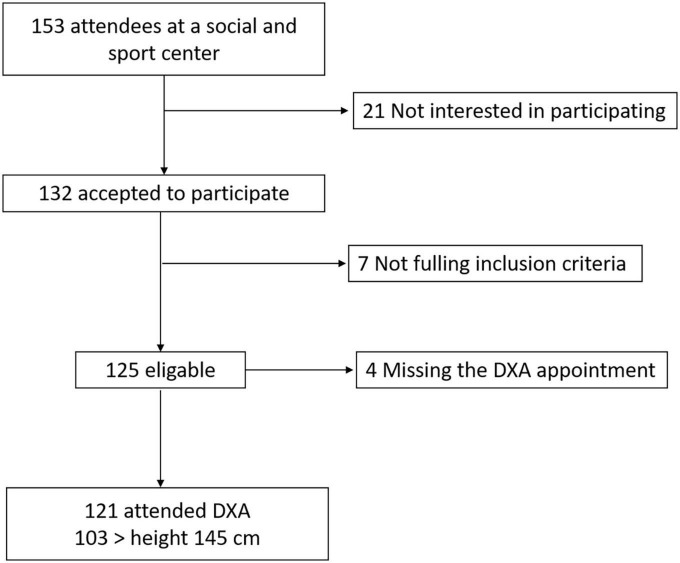
Flow chart of participant recruitment.

The sample size was calculated for a type I error at α = 0.05 and power = 90% (type II error β = 0.10) and an expected Pearson Correlation Coefficient *r* = 0.40, which is considered a moderate correlation, the sample size obtained was 62. The Pearson Correlation Coefficient is frequently used as part of the evaluation of reliability of body composition equations (37). The authorities of the facilities visited wished to include as many participants as possible. The number of participants was scaled up to 132 of these 125 fulfilled the inclusion criteria, 4 did not attend the appointment for DXA examination. In the end, the data of 121 participants was analyzed. Figure 1 presents a flow chart of the participant’s selection process.

### Statistical analysis

The description of the data included means and standard deviations (±sd) for the continuous variables. Categorical data was presented as percentages. DXA BF% estimates were used as reference values to compare BIA and the five different equations that were tested. The normality of the main variable distributions was assessed using the Shapiro Wilkins test. As part of the accuracy evaluation paired *t*-tests were performed to identify differences in BF% estimations between methods. Simple linear regression models were fitted, and the Coefficient of Determination (*R*^2^) and Standard Error of Estimates (SEE) were reported. Lin’s concordance correlation coefficient (CCC) and the 95% confidence interval (95%CI) were obtained. The CCC corresponding graph representing the line of perfect concordance (45-degree line in the Cartesian axes) and the reduced major axis line of the methods being compared were constructed. The reduced major axis regression method has the advantage over simple linear regression to allow error in the measurement of both the independent and the dependent variables (38). This is appropriate considering that DXA body composition measurements have several sources of error. The CCC combines the assessment of precision and accuracy in relation to the perfect concordance line, as observed in the formula (CCC = *r* * C_b): where r is the Pearson Correlation Coefficient, which measures how far the observations deviate from the line of perfect concordance and is considered a measure of precision, and C_b is the bias correction factor that uses measurements of dispersion to estimate the differences of data points with respect to the line of perfect agreement as a measure of accuracy (39). A CCC = 1 indicates perfect concordance between measurements. According to Hinkle et al. (40), the CCC could be classified as follows: 0 ≤ CCC < 0.10 negligible, 0.10 ≤ CCC < 0.39 weak, 0.39 ≤ CCC < 0.69 moderate, 0.69 ≤ CCC < 0.89 strong and 0.90 ≤ CCC very strong. A bootstrap method (1,000 repetitions) was used to obtain 95% confidence intervals of the CCC (95%CI). Additionally, systematic differences (bias) between the tested equations and DXA were evaluated using the Bland-Altman plot, identifying differences between methods and the Limits of Agreement (LoA). The statistical hypotheses tested were considered significant at a *p*-value < 0.05. The statistical analysis was performed using Stata V16 package (Stata Corp. LP, College Station TX).

## Results

A total of 121 older women participated in the study. Their mean age was 73.7 (±5.8), ranging from (65–88) years old. Table 2 presents the anthropometric characteristics of the study group. The mean weight and height were 61.4 kg (±8.8) and 151 cm (±6.0), respectively. The percentage of women shorter than 1.50 was 39.7% and the number of women shorter than 145 cm was 18 (14.9%); the height of 145 cm lies approximately in –1 standard deviation of the height distribution. This subset encompassed 103 women, excluding those with the lowest stature. The mean BMI was 26.9 (±3.6), range (19.4–37.3). None of the women were classified as underweight, and approximately one-fifth were classified as obese according to the WHO criteria. However, using Lipschitz criteria (BMI < 22) 9.9% of the women were underweight and 46.3% (BMI > 27) were overweight or obese.

**TABLE 2:** Anthropometric characteristics and body mass index categories of participating older women (*n* = 121).

Characteristic	
	Mean (± sd)
Age (years)	73.7 (± 5.8)
Height (cm)	151 (± 6.0)
Weight (kg)	61.4 (± 8.8)
Waist circumference (cm)	91.7 (± 9.9)
Hip circumference (cm)	103.6 (± 8.8)
Body mass index (BMI) (kg/m^2^)	26.9 (± 3.6)
BMI, WHO categories	*n* (%)
Underweight (BMI < 18.5)	0 (0)
Normal weight (18.5 < BMI ≤ 24.9)	36 (29.7)
Overweight (25 < BMI ≤ 29.9)	58 (47.9)
Obese (BMI > 30)	27 (22.3)
BMI, Lipschitz categories	*n* (%)
Underweight (BMI < 22)	12 (9.9)
Eutrophy (22 ≤ BMI ≤ 27)	53 (43.8)
Excess weight (BMI > 27)	56 (46.3)

Table 3 presents the mean of BF% estimates by DXA and BIA in the entire group (*n* = 121), and in a subset of taller women (≥ 145 cm). BF% based on DXA was 40.3% (±4.7) while the BIA mean value was 40.9% (±6.1) [difference –0.7, (±3.4)], suggesting an overestimation of BF% by BIA in the study group. The paired *t*-test results indicated a significant difference between measurements (*p* = 0.035) upon the comparison of these two methods. However, in the subset of taller women, the difference between these methods was lower (0.40) and not statistically significant (*p* = 0.228) based on the paired *t*-test results. The SEE was 2.58 percentage points in the entire group and slightly higher in the subset (2.62 percentage points).

**TABLE 3:** Mean percentage body fat (%BF) estimated by DXA, BIA and equations using anthropometric characteristics for the entire group (*n* = 121) and a subgroup of women with heights greater than 145 cm (*n* = 103) and the results of regression models and paired *t-*test.

%BF estimation method/equation	Mean (*SD*) *n* = 121	* *R* ^2^	**SEE	*P*-value for paired *t*-test	Mean (*SD*) *n* = 103	* *R* ^2^	**SEE	*P*-value for paired *t*-test
DXA	40.3 (4.7)	Ref^§^	Ref	Ref	40.3 (4.8)	Ref	Ref	Ref
BIA	40.9 (6.1)	0.703	2.58	0.035	40.7 (6.2)	0.709	2.62	0.228
Deurenberg et al. (54)	43.8 (4.5)	0.463	3.46	<0.001	43.7 (4.6)	0.467	3.55	<0.001
Gallagher et al. (11)	38.2 (4.4)	0.541	3.21	<0.001	38.1 (4.5)	0.538	3.30	<0.001
Woolcott and Bergman height/waist (55)	42.7 (3.6)	0.352	3.81	<0.001	42.5 (3.6)	0.339	3.95	<0.001
Woolcott and Bergman (height^3^/waist, weight) (55)	41.3 (3.4)	0.509	3.31	<0.001	41.2 (3.5)	0.498	3.44	<0.001
Woolcott and Bergman waist/height (55)	41.1 (3.9)	0.515	3.32	0.011	40.9 (3.8)	0.501	3.37	0.074

*R^2^ Coefficient of determination. **SEE Standard error of estimate. ^§^ref, standard reference.

**FIGURE 2:**
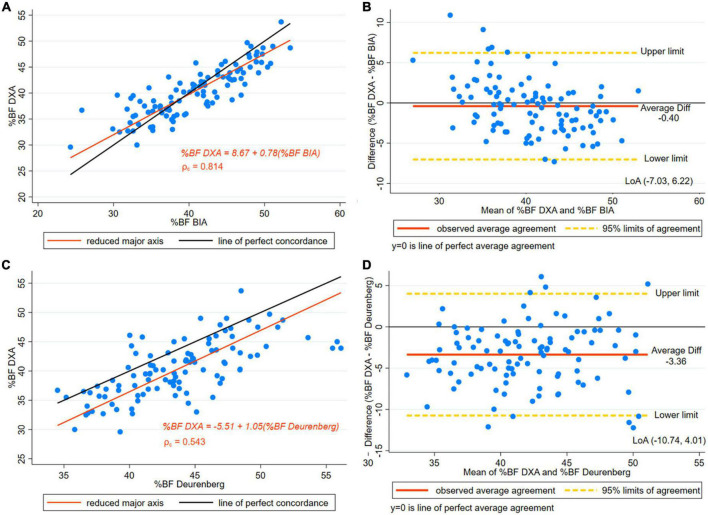
**(A)** Concordance plot of body fat percentage (BF%) estimated by DXA and MF-BIA (multi -frequency InBody 720 equipment). Pearson’s correlation coefficient (r) and Lin’s concordance correlation coefficient (ρ_c_) for women with height > 145 cm. **(B)** The Bland-Altman plot is presented along with the Limits of Agreement (LoA) of BF% estimated by DXA and MF-BIA (Multi -Frequency BIA InBody 720) for women with height > 145 cm. **(C)** Concordance plot for BF% estimated by DXA and Deurenberg’s equation in women with height > 145 cm. **(D)** The Bland-Altman plot is presented along with the LoA for BF% estimated by DXA and Deurenberg’s equation in women with height > 145 cm.

Table 4 presents Lin’s CCC and bias results for DXA and BIA. Satisfactory results were obtained in bias by BIA in both the entire group (C_b = 0.961) and in the subset of the taller women group (C_b = 0.966). The CCC between DXA and BIA was 0.805 and 0.814 in the entire and in the subset groups, respectively. Those CCC are considered to indicate a strong concordance. Figure 2A presents the reduced major axis line and the line of perfect concordance for DXA and BIA of BF% in women with a height over or equal to 145 cm. The data points were distributed tightly along the line of perfect agreement. Moreover, the Bland-Altman plot (Figure 2B) of DXA and BIA results suggest a slight overestimation of BF% (0.40) by BIA, proportional bias was significant and wide LoA (–7.03, 6.22) were observed.

**TABLE 4:** Bias and concordance correlation coefficient for percentage body fat using BIA and equations based on anthropometric measurements in the entire group (121) and a subgroup.

Method/equation	Bias *n* = 121	CCC^a^ (95% CI)	Bias *n* = 103	CCC^a^
BIA^b^	0.961	0.805 (0.750, 0.858)	0.966	0.814 (0.745 0.865)
Deurenberg et al. (54)	0.771	0.525 (0.443, 0.605)	0.795	0.543 (0.423 0.644)
Gallagher et al. (11)	0.902	0.663 (0.576, 0.732)	0.896	0.657 (0.546 0.746)
Woolcott and Bergman (55) height/waist	0.830	0.492 (0.379, 0.603)	0.843	0.491 (0.357 0.605)
Woolcott and Bergman (55) (height^3^/waist, weight)	0.921	0.657 (0.559, 0.744)	0.925	0.653 (0.542 0.742)
Woolcott and Bergman (55) waist/height	0.961	0.637 (0.525, 0.728)	0.959	0.638 (0.517 0.735)

^a^Lind’s concordance correlation coefficient. ^b^Bioelectrical impedance analysis (MF Inbody 720 equipment).

The difference between DXA and Deurenberg’s estimates indicated an overestimated BF% [difference 3.36, (±3.7)] by this equation in the entire group (Table 3), (*p* < 0.001). Similarly, in the subset of taller women, significant differences were found in the paired *t*-test comparison showing an overestimation of Deurenberg estimates (*p* < 0.001). The SEE was higher than three percentage points in the entire group and the subset of taller women.

In both the entire group and the subset of taller women, moderate concordance was observed (Table 4). Figure 2C presents the CCC plot depicting the reduced major axis line and the line of perfect concordance of DXA and the Deurenberg equation. Results of the CCC indicated a moderate concordance. Additionally, in the subset of taller women, the Bland-Altman plot (Figure 2D) BF% shows wide LoA, indicating low precision in the BF% results.

**FIGURE 3:**
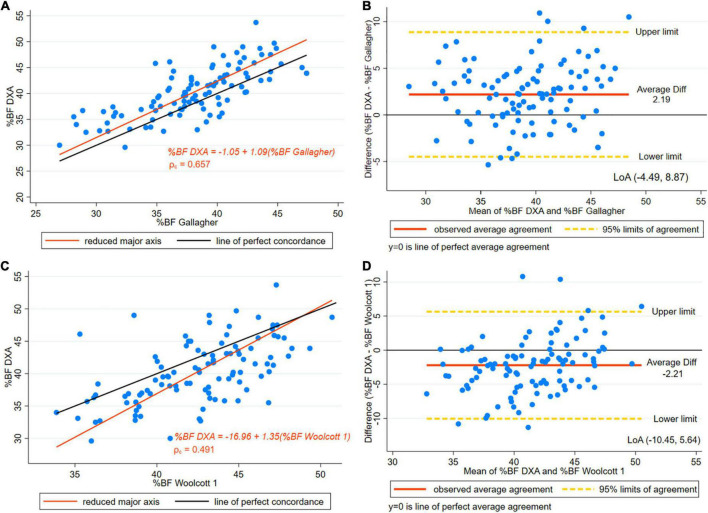
**(A)** Concordance plot of body fat percentage (BF%) estimated by DXA and Gallagher’s equation. Pearson’s correlation coefficient (r) and Lin’s concordance correlation coefficient (ρ_c_) for women with height > 145 cm. **(B)** The Bland-Altman plot is presented along with the Limits of Agreement (LoA) for BF% estimated by DXA and Gallagher’s for women with height > 145 cm. **(C)** Concordance plot for BF% estimated by DXA and Height/Waist ratio Woolcott’s equation (Woolcott equation 1) in women with height > 145 cm. **(D)** The Bland-Altman plot is presented along with the LoA for BF% estimated by DXA and height/waist ratio Woolcott’s equation (Woolcott equation 1) in women with height > 145 cm.

The Gallagher equation underestimated BF% [difference 2.19, (±3.3)] in the women studied; the differences were significant in both the entire group and the subset of taller women (*p* < 0.001) (Table 3). Figure 3A displays the reduced major axis line, and the line of perfect concordance for DXA and Gallagher estimates of BF% in women with a height of more than or equal to 145 cm. The CCC was 0.657, suggesting a moderate concordance between methods (Table 4). The Bland-Altman plot displays the difference of BF% between Gallagher and DXA, where underestimation of BF% was observed (Figure 3B). Bias results showed satisfactory values (Table 4). The findings suggested a slightly higher agreement using Gallagher compared to Deurenberg estimates of BF%.

**FIGURE 4:**
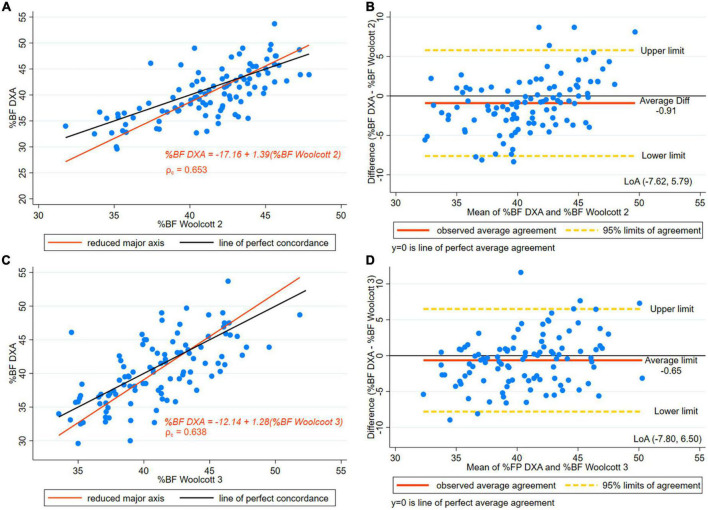
**(A)** Concordance plot of body fat percentage (BF%) estimated by DXA and Height^3^/Waist x Weight Woolcott’s equation (Woolcott Equation 2). Pearson’s correlation coefficient (r) and Lin’s concordance correlation coefficient (ρ_c_) for women with height > 145 cm. **(B)** The Bland-Altman plot is presented along with the and Limits of Agreement (LoA) for BF% estimated by DXA and Height^3^/Waist x Weight Woolcott’s (Woolcott Equation 2) for women with height > 145 cm. **(C)** Concordance plot for BF% estimated by DXA and Waist /Height Woolcott’s equation (Woolcott Equation 3) in women with height > 145 cm. **(D)** The Bland-Altman plot is presented along with the LoA for BF% estimated by DXA and Waist/Height Woolcott’s equation (Woolcott Equation 3) in women with height > 145 cm.

The three Woolcott equations selected showed a statistically significant difference in the paired *t*-test results in the entire sample (*p* < 0.001) (Table 3). However, in the subset of taller women (height ≥ 145 cm), Woolcott’s waist-to-height equation showed no statistically significant difference [difference –0.65, (±3.7), *p* = 0.074]. In the paired *t*-test results for the other two equations the difference remained significant (Table 3). As well, in the Woolcott waist/height equation, in both the subset and the entire group, the *R*^2^ was slightly higher than 0.5. As for the remaining Woolcott equations, both groups had a *R*^2^ of below 0.50, which represents a low coefficient (Table 3). SEE exceeded three points in BF%. A moderate level of CCC was found in all of the three Woolcott equations studied (Table 4 and Figures 3C, 4A, C).

Figure 3D presents the Bland-Altman plot of Woolcott equation using height-to-waist ratio showing a 2.21 percentage points overestimation of BF% compared to DXA and wide LoA (–10.45, 5.64). Figure 4B presents the Bland-Altman plot showing an overestimation of BF% using Woolcott cubic height, waist circumference, and weight equation; the mean difference was around one percentage point (0.91). The graphs displayed LoA (–7.62, 5.79), which appears to be narrower than the LoA obtained by the Woolcott equation using the height/waist ratio. Lastly, Figure 4D presents the Bland-Altman plot of the Woolcott equation using waist/height ratio, a difference of less than one percentage point (0.65) compared to DXA was found. However, proportional bias was significant, and wide LoA (–7.80, 6.50) of BF% were observed in women with heights of 145 cm or higher.

## Discussion

This study aimed to compare the BF% assessment by BIA and five different prediction equations based on anthropometric characteristics, using DXA as the reference method in older women. A significant difference was observed in the mean BF% obtained by BIA and DXA. However, the difference between the mean estimates of BF% with BIA compared to DXA was less than one percentage point. Accordingly, a retrospective study in French adults reported a lack of agreement between BIA and DXA at individual level and good agreement at the population level. Achamrah et al. (32) have suggested that BIA and DXA are interchangeable methods for estimating BF at the population level; nevertheless, at individual level differences were significant. Other studies have reported similar results across categories of BMI, but other authors have suggested that BIA underestimates BF% (30, 37).

The lack of agreement between BIA and DXA body composition estimates may be due to several factors, such as body density and sample selection (age, sex, ethnic group, body density, fat distribution, and body proportions) (41).

A second aim of the present study was to identify the impact of excluding short stature women in the concordance assessment of BIA and DXA. Approximately 40% of the participating Mexican women presented short stature, defined as height under or equal to 150 cm, and 15% presented a height lower than 145 cm. In the current study, in addition to the analysis of the entire group, an analysis was performed in a subset where the shortest women were excluded (those with a height under 145 cm). In the subset of taller women, BIA presented no statistically significant difference in the paired comparisons of BF% with DXA. A good CCC between methods was found, and the SEE was lower than three percentage points. An improvement in the performance of BF% estimates was observed regarding lower mean differences excluding the shortest women.

The effect of short stature in body composition has not been fully elucidated. A high prevalence of short statute has been identified in Mexico (19), Latin-American countries (42), and other areas in the world (43). A study in Mexican adults detected high BF% in short-statute individuals. Short-statute participants (44) with a BMI ≥ 25 presented a 4.2% higher BF% compared to those with normal stature. Furthermore, the influence of short stature on body composition was studied in a group of children using a case-control design matched on age and sex comparing short-stature children with their average-statute counterparts. Differences in body composition were identified, lower fat-free mass was observed in the children with short stature (45). Height is a long-term indicator of growth associated with nutritional status during growing stages. Certain diseases, health behaviors, and socioeconomic conditions may affect height. Genes have a key role in height; recently, the list of genes associated with short stature has increased (46–48).

Short stature is a considered a risk marker for mortality. In a systematic review and meta-analysis of longitudinal studies, a U-shape relationship was observed between height and the risk of death (49). Further studies on the body fat of adults with short stature are warranted to improve the estimation of body composition considering the anthropometric characteristics of this population group.

Studies in individuals under 60 years of age have shown good concordance between BIA and DXA (50, 51). A study in older adults comparing BIA (InBody 720) and DXA found favorable estimates of body composition. However, an equation was developed to lower the error so as to improve the BF% and FFM estimates. In the present study, a multi-frequency BIA equipment was used. The multi-frequency BIA (InBody 720) was found to be superior to a single-frequency BIA (Tanita BC-418) in terms of accuracy in the estimation of fat mass and fat-free mass (52). Results of the PREVED cohort study of the association between body fat and cardiovascular risk found that BIA estimates predict cardiovascular risk better than BMI and waist circumference (53).

In the current study, considering all participants, significant differences were observed between the five anthropometric-based predictive equations and DXA in BF% results. Applying the Deurenberg’s equation (54) led to an overestimation BF% being detected (more than three percentage points). Additionally, the limits of agreement were wide. This equation was derived from a sample of the Netherlands, and the age range of the participants was 7–83 years old, and the prediction formula included BMI. Deurenberg et al. constructed specific equations for both children and adults. It is possible that differences in the age range and ethnicity may contribute to the BF% discrepancies observed between Deurenberg’s equation and the results of DXA for the older Mexican women who were studied. In contrast to our results, a Brazilian study found an adequate prediction of BF% using the Deurenberg’s equation in older women (37). As in the present study, in the Brazilian group, BF% was overestimated with Deurenberg’s equation, and SEE was higher than three percentage points. This error is considered high for clinical practice applications (33).

The Gallagher et al. (11) equation uses 1/BMI, age, and sex for BF% estimates. The results indicated that the predictive equation underestimated BF% in approximately two percentage points compared to DXA in the Mexican older women. Gallagher’s equation was constructed from an international sample including individuals from UK, Japan, and the US. The sample excluded adults with a BMI ≥ 35. In the present study, however, older women with high BMI were included. Similarly to our results, a study in French adults detected a significant difference in the estimated BF% and BF (kg) applying the Deurenberg’s and Gallagher’s equations (33). In contrast, a study in older UK men reported satisfactory results in the validation of Gallagher’s equation for the prediction of lean body mass (31). It is likely that differences in ethnicity and inclusion criteria contributed to the low accuracy of these prediction equations in the Mexican women examined.

In the current study, the Woolcott equation (55) that includes the height/waist ratio overestimated BF% and showed significant difference compared to DXA, and low precision with a large error margin (SEE > 3). These results were similar in the three Woolcott equations that were previously tested. However, in the subset of older Mexican women with a height of more than or equal to 145 cm, the Woolcott equation using the waist / height ratio showed no significant difference to DXA in the paired comparisons. This equation had a moderate concordance according to Lin’s CCC results.

Woolcott and Bergman created (55) body composition prediction equations from the data of NHANES 1999–2004 (*n* = 12,581), and cross-validation was performed with NHANES 2005–2006 (*n* = 3,456). This study applied DXA as reference method. More than 350 different anthropometric, empirical equations were constructed and tested. The best fitted equations were obtained using waist circumference and height and showed better results than the equations based on BMI. In this study, the equation using (cubic height/ waist × weight) had the highest correlation among women in the estimation of total BF%. The results of this equation were close to those of the best anthropometric equation identified in the preset study, which used the waist/height ratio; the former equation performed better than the Deurenberg and Gallagher equations that were based on BMI. This suggests that height and waist circumference may be suitable anthropometric characteristics to be used in equations estimating the BF% in older women. Nevertheless, in the present study, the Woolcott equations that were analyzed showed a SEE > 3 percentage points, and the limits of agreement were also large, showing low precision of the BF% results. Woolcott et al. identified a decline in weight, height, and FFM after the age of 50, additionally, fat mass and waist circumference decreased after age 70. The authors suggested that the lower predicting ability of the different equations analyzed in older age groups may be related with the anthropometric and body composition changes experienced during aging.

The evidence of the validity of body fat estimates through BIA and prediction equations using anthropometry in the older adults is scarce. Some studies in individuals under 60 years of age have shown good concordance between BIA and DXA (50, 51). In older adults, it has been suggested that the use of prediction equations using BIA information improves the validity of body composition estimates. Accurate evaluation of BF% is particularly important considering the high prevalence of obesity and the high burden of NCDs, associated with obesity. Additionally, obese older adults showed higher risk of mortality when they developed infectious diseases compared to those in the normal weight group (56).

In the present study, more than 45% of the Mexican women were overweight or obese based on the Lipschitz criteria for older adults. The Mexican National Health and Nutrition Survey (ENSANUT 2018–19) results indicated that the prevalence of this condition continues to increase (19). It is important to emphasize that there is an increase in obesity prevalence worldwide, which has been described as an epidemic (57). In Mexico, for more than two decades, obesity has been identified as a serious public health problem (58).

Increased obesity prevalence will result in growing of obesity-related chronic diseases. This relationship has been extensively investigated in terms of its effect on disability and mortality among older adults (59).

The impact of obesity on older adults goes beyond their inability to remain independent but also increases the burden on their families, their care givers, and their communities in general (60). Obesity prevention and management programs at the clinical and public health levels for older Mexican people are required.

Additionally, it was found that approximately 10% of the participating women were underweight based on the Lipschitz BMI classification. Low weight is associated with a precarious socioeconomic status, other factors that may favor low weight are a pro-inflammatory state, depressive symptoms, or cognitive disorders (61, 62). Unintentional weight loss or low body-mass index may be an indicator of malnutrition in the elderly because it may reflect energy and nutrient deficiencies, which are difficult to detect in the older adults (63).

## Strengths and limitations

As far as it was possible to investigate, this is the first study that assessed the concordance of BIA (InBody 720) using DXA as a reference method in older Mexican women. Older Mexican women share anthropometric characteristics with women of Latin American and other countries in the world (64). It is important to notice that no significant differences were observed in women taller than 145 cm between BIA and DXA, with the SEE being 2.6 percentage points of the BF%. BIA is a simple technique and available in many settings; thus, the finding of a satisfactory agreement between BIA and DXA supports the use of these devices in the nutritional assessment of older adults; however, improving its precision is desirable considering the large LoA observed. Additionally, the Woolcott predictive equation based on the waist /height ratio showed no significant mean differences compared to DXA when the shortest women were excluded. Utilizing anthropometric measures in order to obtain body composition is useful when resources are limited and DXA is unavailable. The study included women 60 years old and older, active, and living in the community; therefore, the results may not be extrapolated to populations with severe illness and disabilities or those that are institutionalized. There are limitations when using BIA; this method is affected by the hydration status and dehydration is difficult to diagnose in older adults. Additionally, DXA was used as the gold standard, yet there may be errors in estimations of body composition using this technique, regarding body thickness and adiposity, and limitations in the assessment of lean and fat tissue overlying bone structures. Additionally, DXA results may change when using different software or equipment. However, it is frequently used as a reference method in body composition studies and has advantages such as the facts that it is not invasive and that it has good concordance with more advanced techniques in the evaluation of body composition (26).

## Conclusion

In summary, significant differences of BF% estimates were observed between BIA and DXA and between the anthropometric based prediction equations and DXA. In older women who were 145-cm-tall or taller, BIA estimates were closer to the DXA results, and the concordance was good. Additionally, Woolcott’s equation based on the waist/height ratio showed no significant mean difference in BF% estimates from DXA in this group of taller women. Thus, excluding the women with the lowest height decreased the mean difference between methods. The mean difference between BIA and Woolcott’s equation and DXA was less than one percentage point. Nevertheless, the concordance of the Wolcott prediction equation was only moderate. The results indicated that BIA BF% estimates may be more accurate than the five anthropometric-based prediction equations that were tested. The results of this study may assist healthcare professionals who are working with older women in selecting the appropriate methods for BF% estimations.

## Data availability statement

The original contributions presented in this study are included in the article/supplementary material, further inquiries can be directed to the corresponding author.

## Ethics statement

The studies involving human participants were reviewed and approved by the Ethics Committee of the Metropolitan Autonomous University, Unit Xochimilco (Division of Biological and Health Sciences, DCBS/52-17-20) at Mexico City. The patients/participants provided their written informed consent to participate in this study.

## Author contributions

MV-A, MI-C, and MZ-Z: conceptualization and formal analysis. MV-A, IR-C, and MI-C: data curation, supervision, and investigation. MV-A and MI-C: funding acquisition. MV-A, IR-C, AC-S, JF-F, and MI-C: methodology. MV-A, IA-C, IR-C, LM-G, and MI-C: resources. MZ-Z and MI-C: formal analysis. MV-A, MZ-Z, IA-C, IR-C, LM-G, AC-S, JF-F, RG-J, and MI-C: writing—original draft and review and editing. All authors contributed to the article and approved the submitted version.
